# Differentiation of Remedies

**Published:** 1885-06

**Authors:** P. P. Wells

**Affiliations:** Brooklyn, N. Y.


### HOMŒOPATHIC PHYSICIAN,

A MONTHLY JOURNAL OF MEDICAL SCIENCE.

"If our school ever gives up the strict inductive method of Hahnemann, we are lost, and deserve only to be mentioned as a caricature in the history of medicine."—CONSTANTINE HERING.

Vol. V. JUNE, 1885. No. 6.

### DIFFERENTIATION OF REMEDIES.

P. P. WELLS, M. D., BROOKLYN, N. Y.

(*Continued from page 162.*)

These remedies have marked peculiarities in the pains and swellings they excite in the face. Acon. has *crawling pain* with *sense* of *swelling* in the *cheek; pain, as of ulceration*, in the *zygoma;* pain in *one side, with swelling* of the *lower jaw;* pain in the *joint of the jaw* while chewing.

Bell. has severe *cutting pains; pressing, squeezing, tearing, drawing* in the *zygoma; stitch* in the *joint of the jaw*, also while chewing, and into the ear, or from the ear to the chin; *swelling* of the face, with *hardness* and *shootings* therein, in rough weather.

Bry. has *tenseness* in the *skin* of the face and on the *forehead* while *moving the muscles of the face; tearing* and *jerking* from the *zygoma to the temples, aggravated* by *touching; pressure* under the *right malar bone*, disappearing from touching; *pinching* in the *joint of the jaw*—worse from *motion; throbbing* in all parts of the face, to be felt externally; *swelling* of the face—*red, soft, hot, blue* and *brown red*—also only on the *left side*, or from the *nose downward*, or of the *upper half*, especially *over the nose* and *under the eyes*, with *swelling of the lids; hard swelling of the cheek*, near the ear, with burning.

In selecting a remedy for pains or swelling of the face, first note the *kind* of pain. If, as in the case with these three remedies, the pain to be treated is found repeated in the record of several drugs, then compare the concomitant symptoms of the pain with those recorded as concomitants of the pains produced by the several drugs, and also the circumstances which mark the first appearance of each, and which increase or relieve the pain, and then select that drug in the record of which is found the greatest similarity to the record of the case in these particulars. For instance, in Acon. the pains in the zygoma are like *pains of ulceration;* in Bell. the pains in this part are *pressing, drawing, tearing, squeezing;* in Bry., *tearing, jerking,* from the zygoma *to the temples.* Acon. has pains in the joint of the jaw *while chewing.* Bell. has *stitch in the joint,* while chewing, *extending to the ear;* Bry., *pinching* in the joint, made *worse by motion.* In this manner *all* the parts of a case are to be examined and compared before the process of differentiation can be *begun.* And this gives the opportunity for saying that the greatest difficulty in practical homœopathic therapeutics is not in *finding the remedy,* but in gathering the facts of the case, *all* of them, and putting them in such form as will make the required comparison of these with those of the materia medica record *possible,* by which alone the remedy can be found.

Then as to swellings. Acon. has *sense* of swelling *with crawling pain,* and also swelling of the *lower jaw;* Bell., *swelling of the face, with hardness and shootings in rough weather.* In Bry. the swelling is *red, soft, hot,* on the *left side* from the nose downward. By these few examples it will be seen that, though each of these remedies have similar pains and swellings in the face, yet they are accompanied by differences which make their differentiation not only possible but easy.

In the teeth Acon. is characterized by sensation of *looseness,* with *crawling* and *burning* in the *jaws* and *tongue, shootings, pressure* in those of the *left upper jaw, throbbing* pains in *one side, pains* from *mental emotions,* especially *anger; congestive* pains in teeth and face.

Bell. has *convulsions* of the *muscles of the jaws and face* (tetanus); pains of a *rheumatic* character, especially in women and in those who are pregnant; pains from *air currents;* pains which appear *immediately after eating,* which *gradually* increase to a high degree of intensity and as *gradually* disappear; *drawings, shootings;* pains at night, in those of the *right upper jaw* preventing sleep, with acute *burning swelling* of the part and *jerkings* in the teeth, with *drawing* in the ear, or from this downward to a hollow tooth, where it *bores;* better *when* eating, worse *after,* and worst of all at *night;* pain as from *excoriation* while walking in the open air; pain in the *nerve* of the *root* of the tooth in the evening, in bed, and during mental labor; *jerking* and *boring* in the hollow teeth; pains *in the roots* while biting as if *ulcerated*, or as if they would *break; tearing* and *digging* also in hollow teeth, worse when *touched*, especially *by food*, and also from *admission of cold air* to the affected tooth, worse also in the evening; *shootings* sometimes in the ear, or tooth, or face, and also in the night, *after midnight*, on waking; also in hollow upper molars, day and night, followed by swelling of the face; pains in the teeth with *red, hot face*, with *throbbing* in the *head;* sensation as if the teeth were *elongated; swelling of the gums* with *burning* in them, or with chills and fever; *pain in gums* as if *ulcerated* from being *touched; vesicles* on *gums*, which pain as if *burnt; bleeding from hollow teeth.*

Bry. has pain in molars *while chewing; unendurable pain in repose*, especially *in bed*, relieved by *chewing;* pain from taking *warm things* into the mouth; pain from *air* admitted to the tooth; *jerkings* to the ears, with *shooting*, in the evenings in bed, now in upper and now in lower teeth; *drawing* in molars *while* or *after* eating, with feeling of *looseness* or *elongation; tearing, shooting*, even to the muscles of the throat, while eating, and worse from *warm things*, with *tearing* in the cheek and *pinching* in the ear at *night;* pain as if the tooth were screwed in and then torn out, relieved by cold water and going into the open air; pain as from *excoriation* from *cold drinks; after* midnight (3 o'clock) as if *cold air* pressed on *a bare nerve* in a hollow tooth, *relieved* by lying on the *painful* side, and greatly *aggravated* by lying *on the other; looseness* of the teeth, with *pain while chewing and biting*, and *sensation* as if the teeth *were too long*, especially in the morning when waking; gums pain as if *excoriated* and *raw; spongy gums.*

Symptoms of the teeth are chiefly important in treating affections of the teeth themselves. They may at times give light on the selection of the specific for chronic maladies, and sometimes, though less frequently, in acute cases. If any of these be present in either they make a part of that whole we call the "totality," which alone and always determines this choice, and, therefore, are never to be overlooked or neglected. That in the affected and changed life force which determines the character of the pains in the teeth to be *shooting, drawing, tearing, burning*, or of any other kind, and of which we know nothing, except as we meet its effects, is very likely essentially active in modifying the actions of the life forces in other organs and functions, the sum of which constitute the sickness for which we are to find the specific. So far as the symptoms of the teeth disclose this character they become of the first importance.

These remedies act on the mouth, producing symptoms the like of which are found in many sicknesses, especially in acute attacks, and these are often of import in pointing to the specific curative which is about to become an object of search. Acon. has *sensation* of dryness, or *dryness* of *mouth and tongue*, also with *heat*, which *rises* from the *chest* to the *head; crawling, smarting, stitches*, and *burning* of the tongue; brief *paralysis* of the tongue; *trembling, stammering* speech; *excoriating* pain of the *orifices* of the *salivary ducts*, as if they had been corroded; *salivation*, with *pricking* in the tongue.

Bell. has *offensive smell from the mouth, sensation* of great *dryness*, while the mouth is really *moist*, also with very irritable disposition, or with *smarting* and *exfoliation* of the *lips*, with *sticky clamminess* of the *mouth;* great *dryness*, which extends to the *nasal* passages and *throat, constricting the larynx, not permitting* swallowing, and with or without thirst; *froth* before the mouth (epilepsy), bloody, also with *grinding* of the *teeth* and *shaking* of the head; *smells* like *spoiled eggs; salivation* great, with *sticky, thick, white, stringy* saliva; orifices of the salivary ducts *raw* and *excoriated; sticky* mucus, mostly with *dryness* of the mouth; *putrid tasting mucus;* frequent spitting of *sticky* mucus; red inflammatory *swelling* of the *mouth* and *throat; blood* bursting from the *mouth;* tongue *red, cracked, hot*, and *dry; red* on the *edges*, white in the *middle, white* coated, with *sticky, white*, or *yellow* mucous covering; papillæ *dark red, inflamed*, and *swollen; burning* and *smarting* of the tongue, as if there were *vesicles*, and especially when touched, and the whole tongue is painful; *cold* and *dry* sensation; *numbness* of the tongue; *trembling* of the tongue; *paralytic weakness* of the organs of *speech; heaviness* of the tongue; *difficult, stammering* speech, as if intoxicated; *nasal* speech; *loss* of speech.

Bry. has sensation of *dryness* on the *hard palate* and in the mouth *in the morning*, and *between* the upper lip and teeth; great *dryness* of the *mouth, with much thirst*, or *with none*. Great *flow of saliva*, also *frothy* and suds-like; much *spitting* of saliva; *running of saliva* from the mouth; *foul smell* from the mouth; tongue coated *white* or *yellow; dry* tongue; *rough, dark colored; burning vesicles* on the margin; *indistinct* speech from *dryness in the throat.*

It will be noticed that the affections of speech in Bell. and Bry., the record of each of which has embarrassments of this function, are very different in their character, importance, and significance, that of Bell. pointing to paralytic brain affection, as in typhoid fever, or in impending attacks of apoplexy; that of Bry. is more the result of the local condition of the throat, *dryness.* The salivation produced by these two remedies is also different; that of Bell. characterized by likeness to that of epilepsy, and is often accompanied by *dryness* of the mouth. Bry. has copious salivation, which is often frothy and suds-like, with *much* spitting, or it runs involuntarily from the mouth. In contrast with this the saliva of Bell. is found to be thick, sticky, white, and stringy. In this last peculiarity it touches the characteristic salivation of Kali Bi-Chrom. It is rather indicative of local affection of the salivary glands than of any deeper or more important affection, as is the case with that of Bell. With Acon. there is no embarrassment of speech and the salivation is less important, and is accompanied by pricking in the tongue. It has also its characteristic sensation of *crawling* in the mouth, which neither of the others have.

The orifices of the salivary ducts are only affected by Acon. Swelling of the internal mouth is only recorded of Bell. Offensive smell from the mouth is found with Bell. and Bry. It is characterized with Bry. by hawking up offensive tough mucus.

In the throat Acon. has *scratching*, with difficult swallowing. *Sticking, strangling* in the throat, especially while swallowing and speaking; *crawling, burning*, and *fine* sticking and sense of *contraction*, as if from an astringent, in the back part of the throat; inflammation with fever, and *dark red* color of affected part (fauces, soft palate and tonsils), with almost entire inability to swallow, with hoarseness.

The throat with Bell. is *scraped, rough, raw*, especially when touched with the tongue, chewing, or swallowing; *tearing* in left tonsil, especially when swallowing; *burning* in the throat, especially when swallowing food and drinks; *shootings* extending to the ear, or with pain as if swollen, only while swallowing, turning, or feeling the neck, also when not swallowing and when swallowing and breathing; great and suffocating *dryness* in the throat; feeling as if there were a *ball* in the throat which nothing moves; *inflammation* in the throat, of the soft palate, uvula, and tonsils; suppuration of the tonsils—the inflamed parts are covered with a white, tenacious mucus, like a skin; swallowing very painful or impossible, even fluids, which often return through the nostrils; impossible swallowing, with great *aversion to fluids; constant* desire to swallow, with sense of *suffocation* if he does not swallow; *dryness of throat and mouth*

prevents swallowing; *paralytic weakness* of the muscles of deglutition; *spasmodic constriction* and painful *contraction*, with impossibility to swallow the least thing; *strangling* in the throat, with *pressure*, as if from the abdomen; *contraction* of the *œsophagus*.

Bry. has *sensation of dryness*, or great *dryness*, in the throat, especially in the evening; with sensation of *rawness* in *empty swallowing*, worse in a warm room, renewed by drinking; pain in the throat, with difficult swallowing and hoarseness; food and drinks produce choking and will not go down; *pressure* in the throat like a hard body; *stitch* while swallowing, turning the head, and feeling the neck; *scraped roughness* in the back part of the throat; sensation of swelling in the throat, or as if there were an accumulation of mucus, especially while swallowing, also with difficult speech; painful sensation as if the œsophagus were contracted.

The throat symptoms of these drugs are mostly related to sick conditions in which *local* throat troubles are a prominent feature. With Acon. and Bry. these are of an inflammatory nature, and are characterized, Acon. by the *dark* red color of the inflamed surface and by the *sticking, strangling* when swallowing or speaking, or by the *crawling* sensation so common in the actions of this drug on other parts, and Bry. by the *dryness* or *sensation* of dryness; sensation of *rawness* on *empty* swallowing, *worse* in a *warm room; pressure* in throat like a *hard body; sense of contraction* in the œsophagus. With Bell. the range of relation is broader. It has *scraped, rough, raw* sensations; *tearing, burning shootings*. It shares *great* dryness with Bry., and like it has a sensation as of a *ball* (Bry., hard body) in the throat. Its inflammation attacks the hard palate and soft parts of the throat, and these are often covered with *white, tenacious mucus*, in this differing from the inflammations of both its kindred drugs. This peculiarity may suggest a relationship to diphtheria, though it will seldom be found curative of the whole malady. The *paralytic condition* of the *muscles* of the throat is often indicative of important *brain affection* which may find its curative in Bell. The peculiar difficulty of *swallowing fluids*, as compared with that of swallowing solids, will at once suggest a relationship to hydrophobia, which it has cured, thus verifying the truth of our law of curative relationship in a most interesting and convincing manner. This symptom is not found in either of the other drugs. The inflamed throat of Bry. is worse in a *warm room*.

The digestive function and organs associated in it are variously affected by these drugs. To begin with taste. Acon. has *bitter, putrid, insipid, fish-like,* or like *bad eggs; bitter* taste of *all food* and *drink, except water.* Bell. has *loss* of taste; *corrupted, disgusting, nauseous,* also with a clean tongue; *putrid,* either *while* or *after* eating; *weak, sweetish, saltish, acid, bitter; food tastes insipid,* or *too salt; bread tastes and smells sour; milk smells disgusting* and *tastes bitter, acid,* and is rejected.

Bry. has *loss of taste;* taste *flat, insipid, pasty, sweetish, putrid, sickly, disgusting, nauseous, bitter, everything tastes bitter; bitter* or *putrid,* with *offensive breath.*

The modifications of taste are less important as indicia to specific remedies than are many other symptoms of changes in the functions of other organs, and for the reason that taste is changed more or less by almost all drugs, and the changes are so like in so many as to greatly reduce their value as guides to curatives. Still when that which is characteristic of any one is met, *i. e*, a modification not produced by other drugs, it should be carefully noted and have the consideration it merits. Bitter, sour sweet, and others like these common to many drugs, are reduced by this fact to second rank in importance in drug selection. But where, as in the record of Acon., the uncommon *fishlike* taste is met, it is almost certain to be accompanied by other symptoms like those of this drug which will place its selection beyond doubt. The same may be said of the nauseous, disgusting taste, *with clean tongue,* found in the record of Bell., and also of its symptoms. Bread *tastes* and *smells* sour, and milk *smells* disgusting and *tastes* bitter; and also those of the record of Bry.; everything tastes bitter; bitter or putrid taste, with offensive breath.

The appetite is affected by these drugs as it is by so many others. The appetite is reported as *lost* with Acon., with *sour* or *bitter* taste, with *pain in the chest* or in the *hypochondria; disgust for food.* Bell. is also characterized by *total loss of appetite,* while *still feeling hungry;* also with headache, or with *rapid* and *weak* pulse. This is the more noteworthy by reason of its exception to the character of the pulse found so almost universally in cases with predominant Bell. symptoms, full and hard. Bell. has also *aversion* to *meat, acids, beer, coffee,* and *milk.* Bry. has *loss of appetite,* with *empty stomach* and *hunger; aversion* to and *disgust* for *food;* loss of apptite *from swallowing the first mouthful.* It has also *canine* and *morbid* hunger, which compels eating *little* and *often.*

Acon. has *burning* and *unextinguishable thirst,* sometimes for *beer,* which does not agree with the patient. Bell. has *absence*

*of thirst*, or, like Acon., *burning*, *choking*, and *unextinguishable* thirst, with *inability to swallow the least drop*, or with perfect *aversion to all fluids*; drinking with *trembling haste*. Bry. has *thirst* for *cold* drinks; does *not drink often*, but *much* at a time; desire for *wine*, *coffee*, or *acid drinks*. *After eating*, Bell. has *contracting* pain in the stomach; *pinching* below the navel; *drunkenness; drunkenness*, or *internal heat* from *beer*. Bry. has, *after every eating*, especially of *bread*, *eructations*, *pressure in the stomach* and *epigastrium*, with *cutting* in the *abdomen or vomiting; distention of the abdomen*.

The *general* disturbances of appetite, such as its loss or excess, are not of great importance in the selection of specific remedies, as these are met, more or less, as the result of the action of almost all drugs, while special aversions or desires are worthy of a more careful attention from the prescriber. So of other gastric functions which show disturbance by eructations, regurgitations, nausea, vomiting, etc., only that which is *peculiar* in these disturbances is always to be most carefully considered. The eructations of Acon., which are characteristic, are: The mouth is filled with gas, which *tastes* like *spoiled eggs; risings* of *sweetish* water, like waterbrash, with *nausea; empty eructations*, or a *fruitless disposition to them*. Bell. has fruitless *inclination* to *eructations; imperfect* or *suppressed* eructations; also with *loss of appetite* and *vertigo; bitter* eructations, also, *after eating; putrid* eructations; *burning*, *acid*, with rising of *excoriating* fluid; with *strangling*, *heartburn*, *waterbrash*, *hiccough;* with *loss of appetite and dullness of vision*. Bry. has frequent *empty eructations*, especially after *eating;* not after *drinking; empyreumatic*, *burning*, *excoriating* the mouth; *bitter* and *acid* after eating, with rising of *sour* water; *regurgitation* of *mucus*, or of *food*, *after each meal*. The differences of this disturbance, as produced by these three drugs, are sufficiently apparent, and need not to be especially pointed out. The three, it will be noted, produce *waterbrash*. If this be met in a case to be prescribed for, the choice of either, or of some other remedy, must be determined by the *concomitants* of the waterbrash or by the general symptoms, as is always the case where symptoms are met like to those of two or more drugs.

Acon. has *disgust*, *sinking*, *nausea*, *retching*, felt in the *epigastrium* (sometimes, later, *under the sternum*, and then *in the throat*), sometimes while walking in the open air, or it is worse while *sitting* and better *when walking; nausea*, as if from eating *sweets* or *fat; vomiting*, with *nausea*, *thirst*, *general heat*, with *copious sweat* and *urine;* vomiting, of *blood*, of *blood* and *mucus*, of

*green bile*, of *lumbrici;* vomiting of *large masses* of *dark-colored* blood.

Bell. has frequent attacks of nausea *in the forenoon;* disposition to vomit *felt in the throat*, with *bittter* eructations, while walking in the open air; with *disgust*, when *beginning to eat;* with *disgust* and *strangling;* with *great thirst*, *empty retching;* with *yawning, blue face;* at *night*, about *midnight*, with *anxious sweat*.

Bry., *nausea*, mostly with *retching*, especially *after food taken with relish;* in the *morning* on *waking;* also with *empty eructations;* with *bitter taste;* with *copious flow of water* from the mouth, *evenings*, before going to sleep, or in the *morning, after rising; after drinking;* with *weakness;* with *anxiety;* after attempts to *drink* or *sit down; empty retching*, also *evenings, with water and mucus*, like waterbrash; with *coldness of the abdomen*.

Vomiting. Bell. has vomiting in the *evening*, with *vertigo* and *flying heat*, with *copious sweat; sleep after* vomiting; *mucus bilious; acid, watery;* of *food, with diarrhœa;* vomiting of *blood*. Bry. has *vomiting immediately after drinking; after* eating *bread;* of *food*, also *hiccough* and *choking;* of *food* and *bile* immediately *after midnight; bitter*, of *bile* and *water*, especially *immediately after drinking, immediately after eating; mucus* in the *evening;* of *yellowish green mucus; bloody* vomiting; *stitch* in the *left* side of *abdomen* while vomiting.

In connection with this symptom of vomiting, we have the opportunity for remarking on the similarity which cures, and for saying it is not the similarity to any *one* symptom which constitutes any drug a curative for any sickness, but the likeness is to be that of "the totality." It is easy to say when a patient vomits, "Oh! *Ipec.*, *Tart. emet.*, *Ars.*, *Verat.*, etc., these cause vomiting, and, therefore, give *one* of them, no great matter which, and the *law* is responsible for the *cure*." The law is *not* responsible for the cure of any cases so prescribed for. Many cases characterized by vomiting are met which neither of these drugs will relieve. Disappointments of this sort have been a multitude, as disappointments from prescriptions based on other *single* symptoms have been. Such prescriptions will oftener fail of curing than otherwise, because such prescriptions are *not* homœopathic, and it is such as are which cure the vomitings which disregard the routine use of Ipec., Ant-t., etc., and all the other symptoms of such cases as are not like those of these drugs. Vomiting is often distressing and violent where the irritation, which has caused it is far away from the stomach, and the seat of this is revealed by the other members of the group which constitutes "the totality," which will insist on its likeness being sought, found, and given before the cure promised to a faithful compliance with the demands of law can be realized.* An example of this false prescribing is not seldom met in cases of young children who are suddenly and from no known cause attacked with vomiting, which occurs at short intervals, and shows no sign of ceasing spontaneously. The doctor is called. This troublesome symptom which has chiefly alarmed friends, and called him to the side of the sufferer, occupies his whole vision. Diagnosis, "*disordered stomach*;" give Ipec., Ant-t., or Ars. The obstinate plague won't stop. The "disordered stomach" is all the doctor sees, and he asks himself what has caused the derangement? He goes over all the child has eaten, and all his known exposure to causes of gastric disturbance, and possibly lights on an article of diet he imagines to have done the mischief. Well, now he has found a clue to the way out of this difficulty. He knows, or thinks he knows, that *Nux vom.* cures the maladies so caused, vomiting included. He gives his specific for errors of diet, and his patient is no better, and now the doctor is puzzled. If he had had the skill and the carefulness so needful to the gathering of the neglected "totality" of symptoms, and had employed them as he should have done, he might have discovered that *in the brain*, and not in the stomach, is the *fons et origo* of all his and his patient's trouble. Such cases are not uncommon, and if recognized and properly prescribed for *in the beginning* are easily cured. If paltered with as "disordered stomachs," and get Ipc., etc., or Nux v. etc., they certainly die. They have had their *vomiting* attended to. They have had no *homœopathic* prescribing.

[TO BE CONTINUED.]

---

* It happened to the writer some years ago to see, in consultation with a neighbor, a case which he, the neighbor, said was a *gastromatacia*. He had been trying for near a week, with the whole train of so-called "emetics," to stop the vomiting of the child (about five years old), but this was obstinate and would not stop. This obstinacy was responsible for the diagnosis, "*softening of the stomach*." The child was found wholly unconscious. Could not be roused by any endeavor to this end. Face pale, cool, damp; eyes staring, pupils largely dilated; paralysis of one side, with increasing disposition to spasms of the voluntary muscles. This was the sight presented to the consulting visitor as he entered the room where the sick child lay. The first glance told the story, and the consultant said to the attendant, "Doctor, you have a case of water in the brain." He saw it *when told!* and threw up both hands, and exclaimed, "My God! *how could I have been so blind!*" But he had been, and was so blind. *Moral:* The man who can see but one thing at a time should never attempt to practice Homœopathy. This worthy man had mistaken his calling, and his patient died.

